# Editorial: Brain and leptomeningeal metastases in lung cancer

**DOI:** 10.3389/fonc.2022.1115126

**Published:** 2023-01-23

**Authors:** Tiziana Vavalà, Jessica Menis

**Affiliations:** ^1^ Azienda Ospedaliero Universitaria (AOU) Città della Salute e della Scienza, Department of Oncology, SC Oncologia 1, Torino, Italy; ^2^ Section of Oncology, Department of Medicine, University of Verona School of Medicine and Verona University Hospital Trust, Verona, Italy

**Keywords:** non-small cell lung cancer, brain metastases, treatment, radiotherapy, lung cancer

(NSCLC) and its incidence is expected to further increase thanks to larger screening, better imaging tools and longer patient survival by more efficacious systemic target and immunological therapies.

As CNS metastases are associated with a negative impact on quality of life and survival, improving their management has become more and more important, requiring stronger management recommendations as well as dedicated randomized trials, including patients with untreated and/or unstable, or even all, CNS metastases that have been usually excluded from most clinical trials. Considering evolution in local approaches (such as stereotactic radiation therapy) there is high need of comprehensive treatment strategies promoted by multidisciplinary brain metastasis tumor boards.

Brain metastasis (BM) is one of the most common failure patterns of stage IIIA-N2 NSCLC after complete resection (Sun et al). Sun et al. developed a predictive nomogram based on clinicopathological factors (non-squamous histology, bronchial or perineural invasion) and treatment approaches (adjuvant chemotherapy) that could select patients at high risk who may benefit from prophylactic cranial irradiation (PCI).

The optimal treatment of CNS metastases and the best order of systemic and local therapy is still not precisely defined. The relevance of an integrated and shared approach to patients with brain metastases is crucial and was highlighted in the retrospective study by Liu et al. on 160 NSCLC patients with brain metastases, that showed a better survival by systemic treatment before local treatment even when used to relieve clinical symptoms.

Local treatment by RT, either alone or combined with surgery and systemic therapies, is a cornerstone of brain metastases management. Mantovani et al. provided an overview (literature data and possible technical solutions) of the many modern RT options available in different clinical scenarios (single lesion, oligo and poly-metastasis, leptomeningeal carcinomatosis).

Still, new predictive biomarkers or imaging tools are under development so to identify patients with better or poorer outcome. Guo et al. evaluated the significance of combination of the magnetic resonance spectroscopy parameters (MRS) and systemic immune inflammation index (SII) in patients with brain metastases from NSCLC treated with stereotactic radiotherapy. The C-SII score was more accurate for predicting clinical outcomes of NSCLC patients with brain metastases who underwent stereotactic radiotherapy.

Focusing on systemic therapies, major efficacy improvements have been achieved in the last years thanks to target agents and immunotherapy but their impact on CNS control as well as their combination with RT still needs further research efforts (Pierret et al.). Some data suggest that immunotherapy compounds are able to cross the blood-brain barrier, resulting in local tumor microenvironment modification but mechanism of intracranial response still merit focused investigation. Moreover, pivotal clinical trials that demonstrated impressive efficacy, often did not include patients with active, untreated brain metastases or leptomeningeal carcinomatosis. Despite important limitations, some real-life studies have described a maintained efficacy of immunological approaches also in less selected patients with untreated or symptomatic brain metastases. Nevertheless, neurological complications have still to be evaluated and carefully monitored.

In this context an increasing attention should be directed at understanding the unique biology of CNS metastases and the molecular basis of intracranial progression since this could lead to new treatment options. In particular, attempts to identify potential biomarkers like the analysis conducted by Peng et al. are fundamental. Nuclear factor of activated T cells (NFAT) c1 and c3 were expressed during NSCLC and BM development and regulated interleukin-11, cadherin 5 (CDH5) and C-C motif chemokine 2 (CCL2).

Finally, among CNS metastases, leptomeningeal metastasis is an even more severe and challenging complication, particularly in EGFR-mutated NSCLC. The retrospective study by Li et al., on fifty-three EGFR mutated NSCLC with leptomeningeal metastasis aimed to evaluate gene mutations, treatment strategies, and clinical outcomes of combination therapy. Treatment with Osimertinib was confirmed to be efficacious in prolonging survival.

In conclusion, the aim of improving the understanding of CNS metastasis biology as well as the best multimodal integrated systemic and local treatments for these patients in the context of larger clinical trials is to lastly obtain a prolonged survival, limiting neurological deterioration in order to protect the best possible quality of life.

## Author contributions

TV and JM contributed equally to the design, implementation and to the writing of the manuscript. All authors contributed to the article and approved the submitted version.

